# Preface

**Published:** 1891

**Authors:** P.P. Wells

**Affiliations:** 158 Clinton Street, Brooklyn, N. Y.


### PREFACE.

---

This book was originally begun as an ordinary paper to be contributed to the pages of THE HOMŒOPATHIC PHYSICIAN, but in preparing it it grew to such large proportions that I finally concluded to publish it as a book. It was, therefore, withheld from the pages of that journal for several years.

It had remained in my desk for so long a time that I had forgotten its contents. I have now decided to present it to THE HOMŒOPATHIC PHYSICIAN for publication in such manner as seems to its editors best.

It represents my own experience during fifty years of practice. I have not undertaken to make a complete treatise upon Intermittent Fever—not at all. It is only the record of my own thoughts and experience during these fifty years. It took me thirty years to learn how to examine a case for prescription. For upon the proper getting of the picture of the condition of the patient depends the success in treating it.

I have cured about fifty cases of intermittent fever in succession upon the *first* prescription of a *single* remedy. I think this a sufficient evidence that the law of similars and the totality of the symptoms—which is the principle upon which I have practiced—is the correct one. I think the reader may be assured that there is no more difficulty in treating intermittent fever than in treating any other disease. By strictly following this principle any one can cure ague.

The Repertory which is added will be recognized as the work of the immortal Bœnninghausen. I translated it, not knowing that Dr. Augustus Korndœrfer, of Philadelphia, had already published a most excellent and accurate translation.

P. P. WELLS.

158 Clinton Street, Brooklyn, N. Y., May, 1891.

